# Pre-colectomy location and TNM staging of colon cancer by the computed tomography colonography: a diagnostic performance study

**DOI:** 10.1186/s12957-021-02215-4

**Published:** 2021-04-15

**Authors:** Yadong Zhou, Zhiwei Han, Fafu Dou, Tao Yan

**Affiliations:** 1 Department of Gastrointestinal Surgery, 3201 Hospital, Hanzhong, 723000 Shaanxi China; 2 Department of Radiology, Air Force Medical University, Xi’an, 710032 Shaanxi China; 3 Department of Radiology, Xian XD Group Hospital, Xi’an, 710077 Shaanxi China

**Keywords:** Colonoscopy, Local staging of colon cancer, Tumor location, Treatment plan, Virtual colonoscopy

## Abstract

**Background:**

The Chinese Society of Clinical Oncology guidelines 2018 and the recent update of that (version 2020) recommends accurate examination before major treatment for decision(s) in cases of colon cancer. Also, the difficulty in the identification of the lesion during colectomy may lead to resection of a wrong segment of the colon or a more extensive resection than planned. Accurate pre-colectomy local staging of colon cancer is required to make decisions for treatment of colon cancer. The objective of the study was to evaluate the diagnostic performance of the computed tomography colonography (CTC) for pre-colectomy tumor location and tumor, node, and metastasis (TNM) staging of colon cancer.

**Methods:**

Data of preoperative colonoscopies, CTC, surgeries, and surgical pathology of a total of 269 patients diagnosed with colon cancer by colonoscopy and biopsy and underwent pre-colectomy location and TNM staging by CTC were collected and analyzed. The consistency between the radiological and the surgery/surgical-pathological for location and TN stages of colon tumor were estimated with the weighted kappa or kappa coefficient (*κ*) at 95% confidence interval (CI).

**Results:**

CTC detected 261 (93%) and colonoscopy detected 201 (72%) correct locations of tumors. Sensitivity and accuracy of CTC for detection of location of colon tumors were 100% and 92.58% (*κ* = 0.89; 95% Cl: 0.83–0.95). 72.48% sensitivity, 90.64% specificity, and 83.57% accuracy were reported for CTC in differentiation of tumors confined to the colon wall (T1/T2) from advanced tumors (T3/T4) (*κ* = 0.69, 95% Cl: 0.51–0.75). 81.01% sensitivity, 89.11% specificity, and 83.93% accuracy of CTC was reported for differentiation of tumors between low–intermediate risk and high risk (*κ* = 0.68, 95% Cl: 0.53–0.75). 69.31% sensitivity, 66.15% specificity, and 67.14% accuracy of CTC were reported for N staging of tumors (*κ* = 0.41, 95% Cl: 0.59–0.69).

**Conclusions:**

CTC has high diagnostic parameters for pre-colectomy location and T staging of colon tumors except patients of colon cancer who received neoadjuvant chemotherapy.

**Level of Evidence:**

III.

**Technical Efficacy Stage:**

2.

**Supplementary Information:**

The online version contains supplementary material available at 10.1186/s12957-021-02215-4.

## Background

The computed tomography (CT) is used in the clinical practice for detection of tumor, node, and metastasis (TNM) staging of colon cancer because of the development of multidetector CT scanners and multiplanar reconstruction software but there are different results reported in the literature at different times for TNM staging of colon cancer [1]. The computed tomography colonography (CTC) or virtual colonoscopy is effective for the detection of colon cancer [2] and is used for the examination of the whole colon [3, 4]. It also allows the identification and examination of lesions of the colon for local staging of cancer, which is difficult in conventional CT [4]. The prospective studies [4–6] and comparative studies [7–9] have evaluated CTC in the local staging of colon cancer and reported promising results with accuracies of 78–92%. The prospective studies [5, 6] and a comparative study [7] on the Italian population, a comparative study on the Brazilian population [8], and a comparative study on the North American population [9] have a small sample size. The prospective study on the Spanish population [4] is a single-center study and is performed by only one radiologist. The prospective study [5] included stenotic tumors, the prospective study [6] included rectal cancer with colon cancer, and a comparative study [7] included stenotic tumors and rectal cancer with colon cancer. These may cause bias because the anatomy of the rectum and colon are different. Also, magnetic resonance imaging is the gold standard for rectal cancer diagnosis [4].

For locally advanced, amenable to resection, nonmetastatic colon cancer (T1N0 to T4bN4), colectomy or endoscopic surgery followed by chemotherapy is recommended, and for those amenable to resection, metastatic colon cancer neoadjuvant chemotherapy and colectomy with/without chemotherapy are recommended by the Chinese Society of Clinical Oncology guidelines 2018 [10] and the recent update of that (version 2020) [11]. Also, the difficulty in the identification of the lesion during colectomy may lead to resection of a wrong segment of the colon or a more extensive resection than planned [12]. Accurate pre-colectomy local staging of colon cancer is required to make decisions for treatment of colon cancer [4]. Also, the Chinese Society of Clinical Oncology guidelines 2018 [10] and the recent update of that (version 2020) [11] recommends accurate examination before major treatment decisions in cases of colon cancer. The ESMO consensus guidelines [13], the SEOM clinical guidelines 2018 [14], and the Chinese Society of Clinical Oncology guidelines 2018 [10] recommend pre-colectomy chest/abdominal/pelvic CT for diagnosis and staging of colon tumor. CTC can perform colorectal cancer screening in asymptomatic patients, thus serving as another screening method. However, because CTC is inferior in detecting advanced colorectal neoplasms, the technique should not replace the optical colonoscopy, which remains the gold standard.

The objective of the retrospective analysis of the cross-sectional study was to evaluate the diagnostic performance of CTC for pre-colectomy location and TNM staging of colon tumors considering the results of the surgeries and surgical pathology as the reference standard.

## Methods

### Study population

**Fig. 1:**
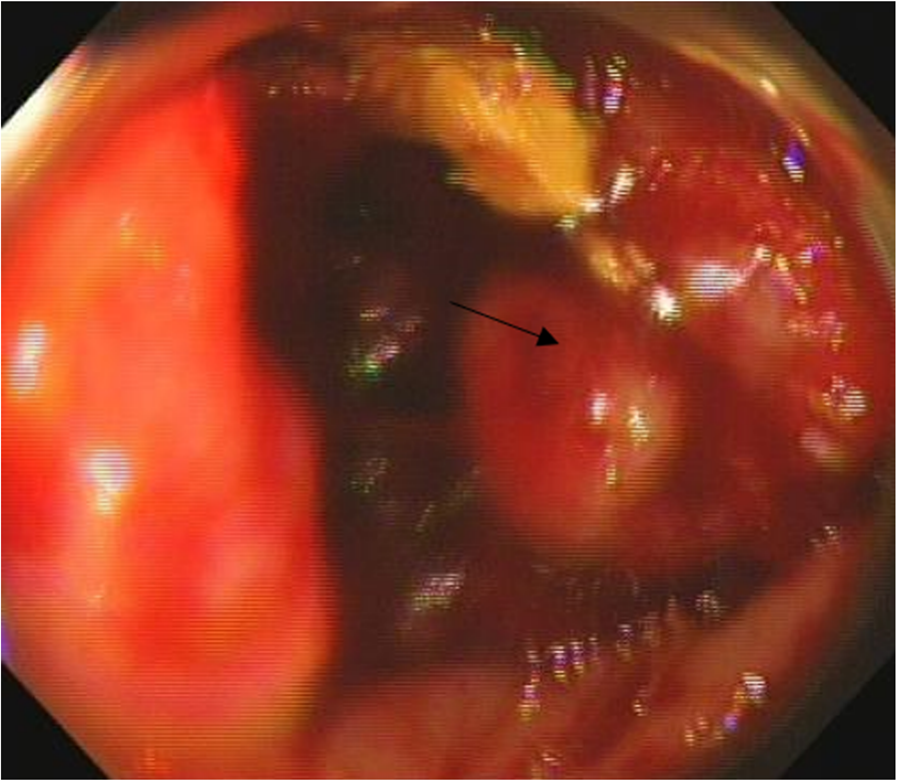
Colon cancer visualized by colonoscopy. The black arrow indicates cancer tumor

**Fig. 2:**
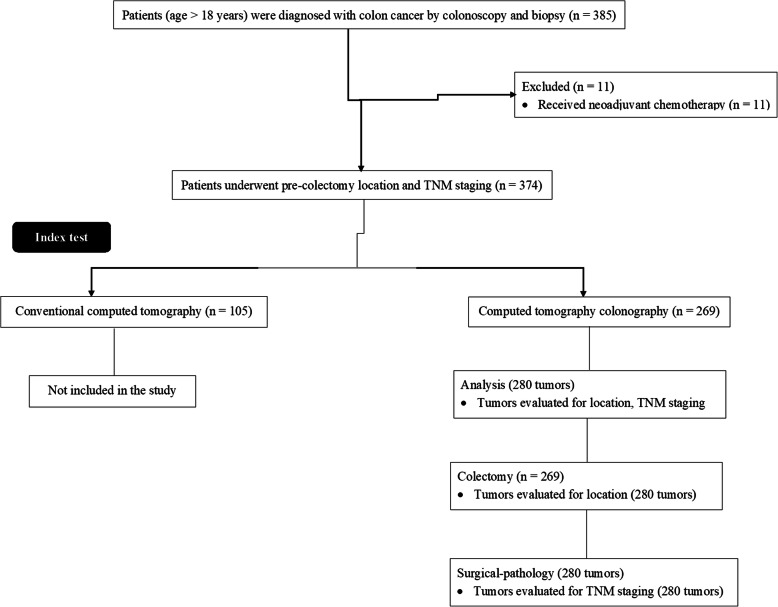
The flow diagram of the management of colon cancer of patients

From 15 January 2018 to 14 July 2020, a total of 385 patients (age > 18 years) were diagnosed with colon cancer by colonoscopy (Fig. 1) and biopsy at the 3201 Hospital, Hanzhong, Shaanxi, China; the Air Force Medical University, Xi ’an, Shaanxi, China; and the Xian XD Group Hospital, Xi ’an, Shaanxi, China. Among 385 patients, 105 patients underwent conventional CT for location and TNM staging and 11 patients received neoadjuvant chemotherapy. Therefore, data of these patients (*n* = 116) were excluded from the analysis. Data of clinical conditions, pre-colectomy colonoscopy and CTC examinations, surgeries, and surgical pathology of a total of 269 patients diagnosed with colon cancer by colonoscopy and biopsy and underwent pre-colectomy location and TNM staging by CTC were collected from the institutional records of patients and analyzed. The flow diagram of the management of colon cancer of patients is reported in Fig. 2.

### Bowel preparation

A low-fiber diet for 2 days followed by a clear liquid diet for 1 day was instructed to all patients before CTC examinations. Fecal tagging was done with 8 mL of oral diatrizoate meglumine and diatrizoate sodium solution (Gastrografin®, Bracco Diagnostic Inc., Monroe, NJ, USA) at every meal (maximum 3 times/day) for 2 days before CTC examinations. A rectal enema of sodium phosphate (250 mL, Fleet, C.B. Fleet Company, Inc., USA) was given immediately before CTC examinations [4]. Bowel preparation was performed under the supervision of radiologists and colorectal surgeons.

### Air distension

In the lateral position of the patients, the gentle air was insufflated manually (balloon-tipped rectal tube) by radiologists (a minimum of 10 years of experience) of institutes before CTC examinations.

### The computed tomography colonography

Patients were examined on a 64-slice multidetector CT scanner (Somatom Sensation 64, Siemens Healthcare, Erlangen, GmbH, Germany) with thin (1–2 mm) reconstruction and multiplanar reformation (MPR) images. To study the colon thoroughly, CTC was performed after air distension of the colon in both supine and prone states. The first acquisition of an abdominal CT of patients was evaluated in the prone position without contrast and at low doses of radiation. The second thoracic and abdominal acquisition of patients was evaluated in the supine position after intravenous injection of 1.5 mL/kg iodinated contrast agent (Iomeron® 400, Bracco U.K. Ltd., Buckinghamshire, United Kingdom) at 3 mL/s injection rate [15]. The technical parameters of CTC for TNM stage detection of colon cancer are reported in Table 1 [4].

**Table 1:** The technical parameters of the computed tomography colonography for location of tumor and tumor, node, and metastasis (TNM) stage detection of colon cancer

Position	Part of body	Contrast agent	Collimation	kV	mAs	Thickness of slice (mm)	Spacing between slices (mm)	Delay (s)
Prone	Abdomen and pelvis	No	64 × 0.625	120	30	2	1	–
Supine	Thorax	Yes	64 × 0.625	120	Automatic modulation 120	3	1.5	35
Supine	Abdomen and pelvis	Yes	64 × 0.625	120	Automatic modulation 180	2	1	70

All CTC data were transferred to work station equipped with dedicated CTC software (Siemens Healthcare, Erlangen, GmbH, Germany). All CTC examinations were performed by two radiologists (a minimum of 10 years of experience in abdominal CT) of institutes.

### Image analysis

**Fig. 3:**
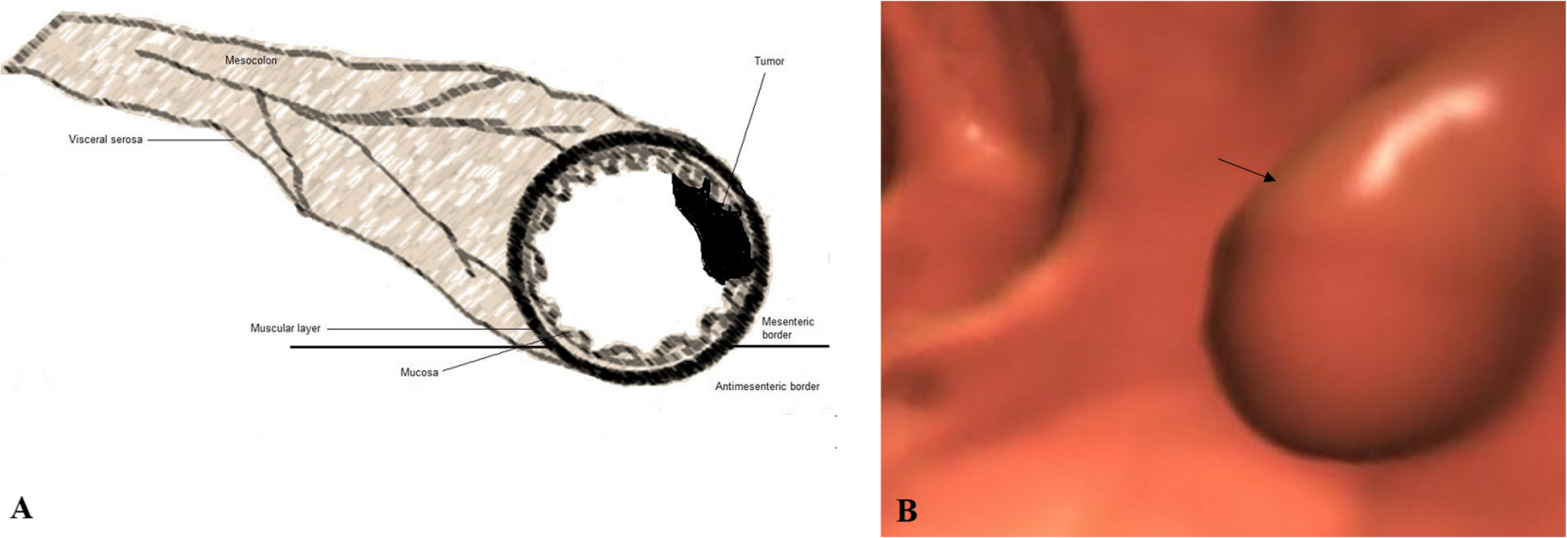
T2 stage: a thickened walled tumor without signs of serosal invasion or pericolonic fat invasion. **a** Schematic representation. **b** The computed tomography colonography. The black arrow indicates cancer tumor

**Fig. 4:**
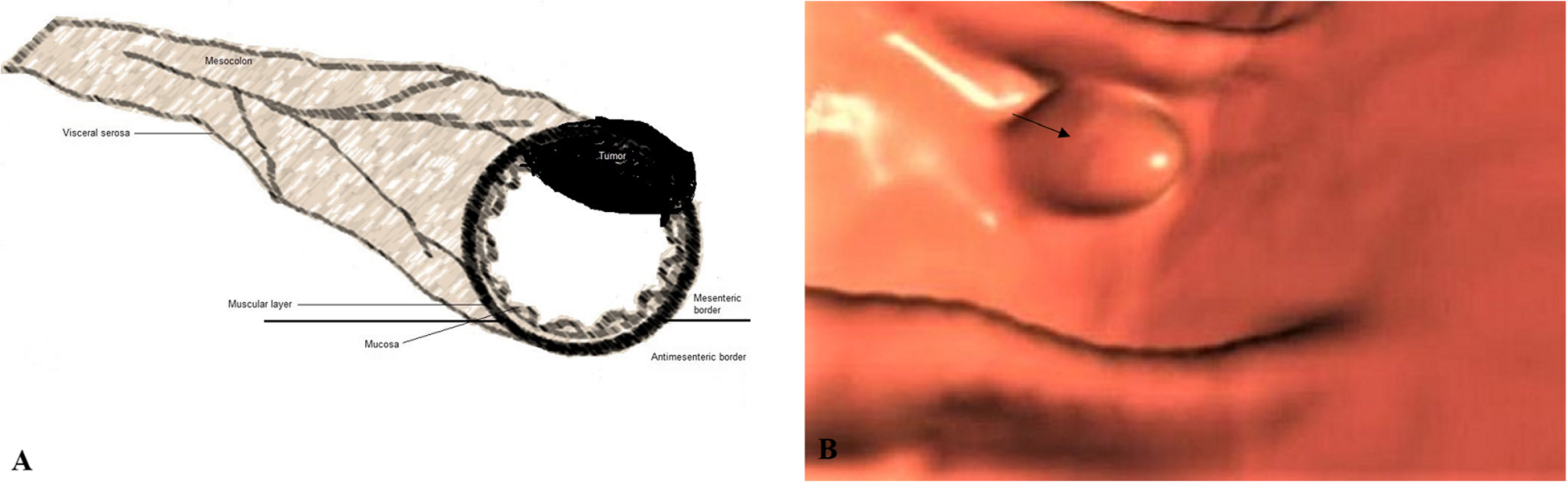
T3 stage: A tumor with pericolonic fat invasion. **a** Schematic representation, **b** The computed tomography colonography. The black arrow indicates cancer tumor

**Fig. 5:**
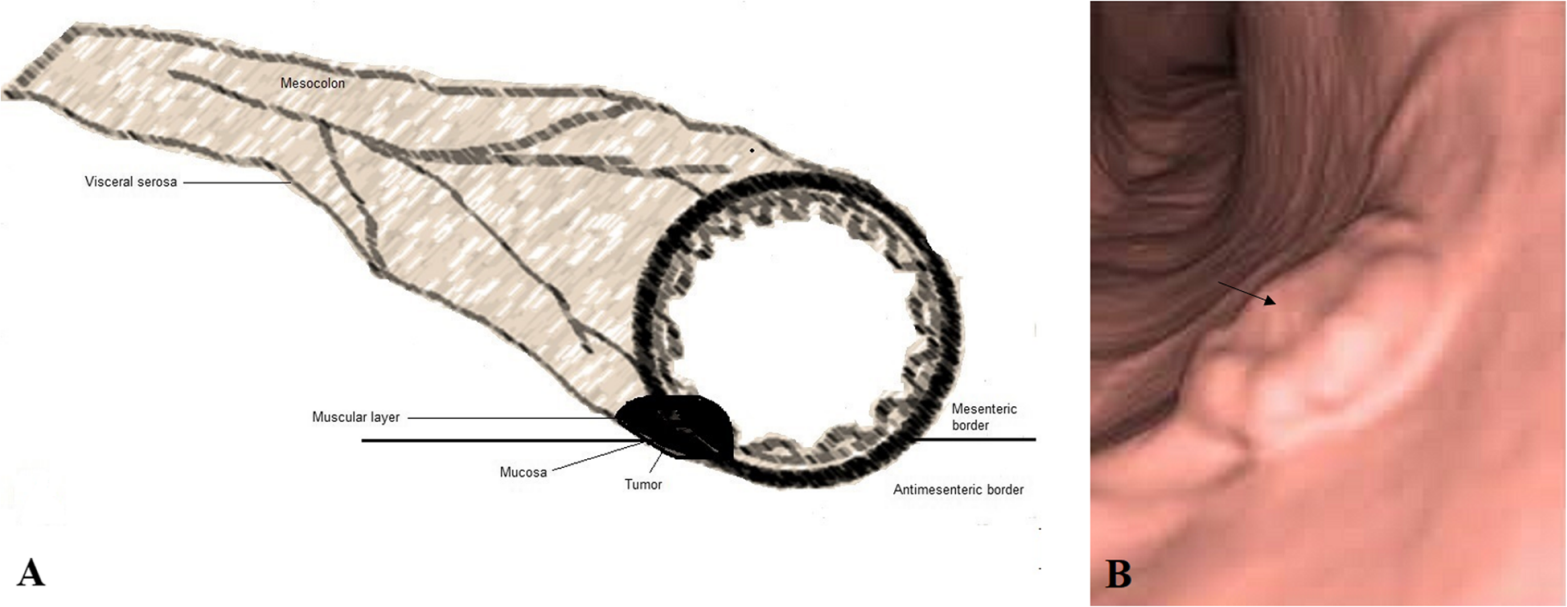
T4a stage: a tumor invading the visceral serosa. **a** Schematic representation. **b** The computed tomography colonography. The black arrow indicates cancer tumor

**Fig. 6:**
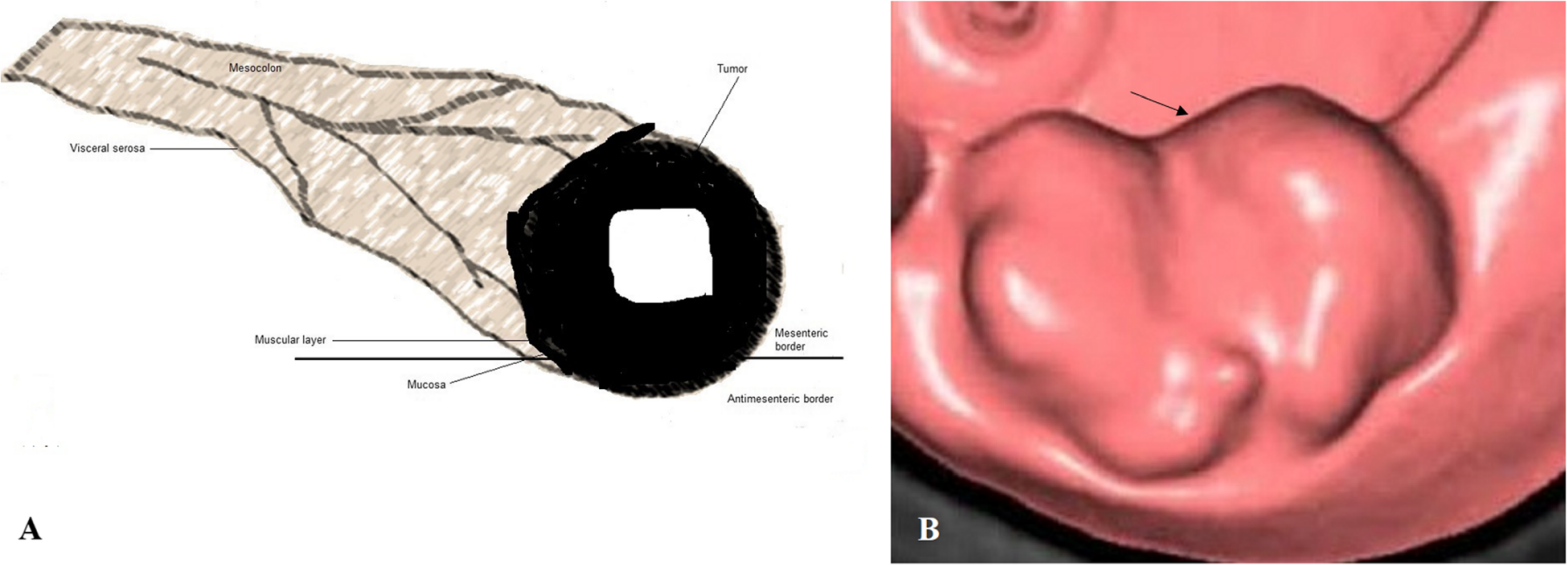
T4b: a tumor with invasion of adjacent organs. **a** Schematic representation. **b** The computed tomography colonography. The black arrow indicates cancer tumor

**Fig. 7:**
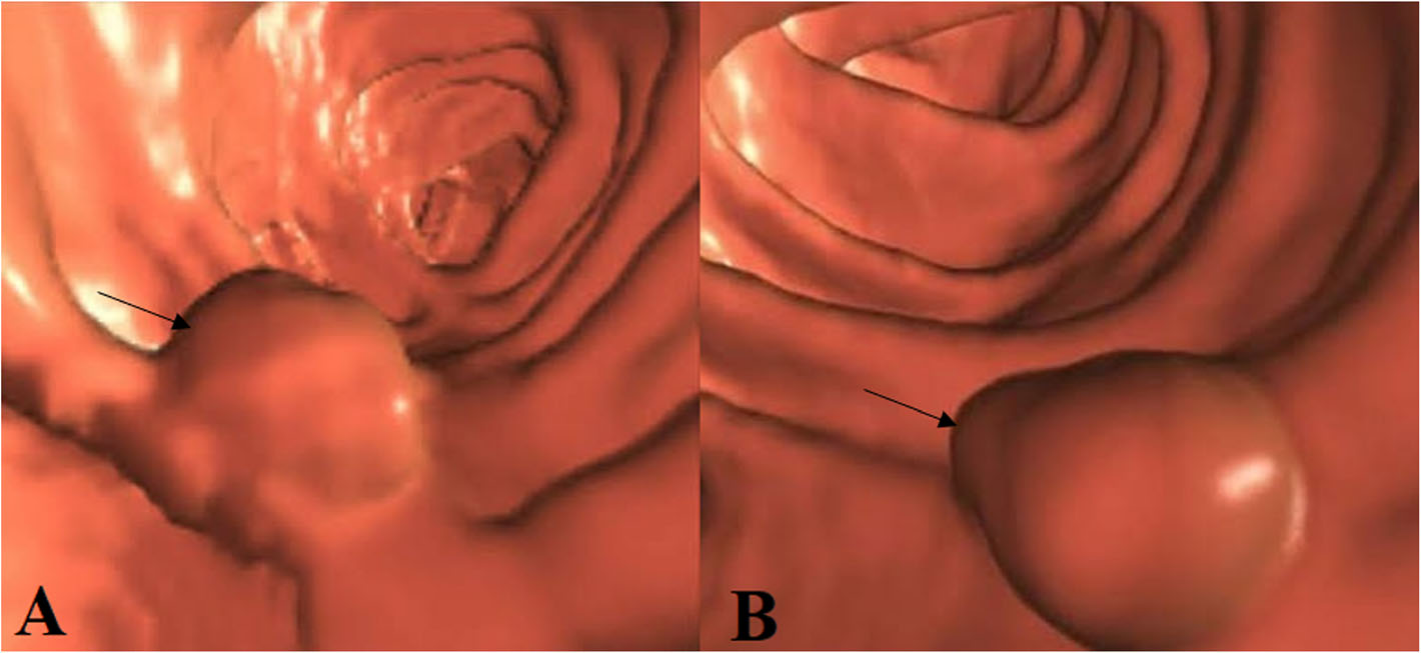
T3 stage. **a** The computed tomography colonography image of T3ab: T3 stage tumor with less than 5 mm extension beyond the muscularis propria. **b** The computed tomography colonography image of T3cd: T3 stage tumor with 5 mm or more extension beyond the muscularis propria. The black arrow indicates cancer tumor

The location (caecum, hepatic flexure, splenic flexure, ascending colon, transverse colon, descending colon, or sigmoid colon), size (thickness and length), the colonic circumference involvement (≥ 50%/ ≥ 180 ° or < 50%/ < 180 °), pericolonic invasion of fat (quantified in mm in the plane transverse to the colonic wall), signs of visceral serosa invasion (linear or nodular thickening of visceral serosa in contact with the tumor, or colonic perforation), and invasion of adjacent organs were analyzed for each tumor. From the features of CTC, the T stages of tumors were classified as follows: 1. T1/T2 stage: a thickened walled tumor without signs of serosal invasion or pericolonic fat invasion (Fig. 3), 2. T3 stage: a tumor with pericolonic fat invasion (Fig. 4), 3. T4a stage: a tumor invading the visceral serosa (Fig. 5), 4. T4b: a tumor with invasion of adjacent organs (Fig. 6) [4]. Tumors were also classified as 1. Low-risk tumor: T1/T2 stage, 2. Intermediate-risk tumor: T3 stage tumor with less than 5 mm extension beyond the muscularis propria (T3ab; Fig. 7a), and 3. High-risk tumor: T3 stage tumor with 5 mm or more extension beyond the muscularis propria (T3cd; Fig. 7b) and T4 tumors [16, 17]. The N stages of tumors were classified as follows: N−: no nodes involvement and N+: one (N1) or two (N2) nodes involvement. If lymph nodes were enlarged more than 9 mm in the *x*-axis, with heterogenous contrast enhancement in nodes bigger than 5 mm, had irregular borders, or had clusters of more than 3 nodes, then it was considered that lymph node was involved [4]. Image analysis was performed by two radiologists (a minimum 10 years of experience in abdominal CT) of institutes. Radiologists evaluated images by consensus.

### Colectomy

According to the location and tumor stage findings of CTC examinations and the clinical conditions of the patients, the colectomy was considered for patients. Surgeries were performed by the two colorectal surgeons (a minimum of 10 years of experience) of institutes.

### Surgical pathology

Histopathological examinations of the surgical specimens were done according to the American Joint Committee on Cancer the eighth TNM classification [18]. Pathologies were performed by a pathologist (a minimum of 3 years of experience; unaware of CTC findings) of institutes.

### Diagnostic parameters

Sensitivities, specificities, and accuracies for CTC and colonoscopies were defined as per Eqs. 1, 2, and 3:

1 $$ \mathrm{Sensitivity}=\frac{\mathrm{TP}}{\mathrm{TP}+\mathrm{FN}} \times 100 $$

2 $$ \mathrm{Specificity}=\frac{\mathrm{TN}}{\mathrm{TN}+\mathrm{FP}} \times 100 $$

3 $$ \mathrm{Accuracy}=\frac{\mathrm{TP}+\mathrm{TN}}{\mathrm{TP}+\mathrm{TN}+\mathrm{FP}+\mathrm{FN}} \times 100 $$

where

TP: True positives: Detected by index test and detected by surgery/surgical pathology.

TN: True negatives: Not detected by index test and not detected by surgery/surgical pathology.

FP: False positives: Detected by index test but not detected by surgery/surgical pathology.

FN: False negatives: Not detected by index test but detected by surgery/surgical pathology.

### Beneficial score analysis

Beneficial score analyses to perform colectomies for CTC and colonoscopy were evaluated as per Eq. 4 [19]. The higher the beneficial score, the easier will be the colectomy at a low level of diagnostic confidence for the colorectal surgeons. The level of diagnostic confidence was determined by colorectal surgeons for each tumor and it is variable (from 0 to 0.99).

4 $$ \mathrm{Beneficial}\ \mathrm{score}=\frac{\mathrm{Correct}\ \mathrm{location}\ \mathrm{of}\ \mathrm{tumors}}{\mathrm{Tumors}\ \mathrm{were}\ \mathrm{excised}\ \mathrm{surgically}}-\left(\frac{\mathrm{Numbers}\ \mathrm{of}\ \mathrm{tumors}\ \mathrm{in}\ \mathrm{which}\ \mathrm{mistake}\ \mathrm{did}\ \mathrm{for}\ \mathrm{location}\ \mathrm{by}\ \mathrm{one}\ \mathrm{or}\ \mathrm{more}\ \mathrm{contiguous}\ \mathrm{segment}}{\mathrm{Tumors}\ \mathrm{were}\ \mathrm{excised}\ \mathrm{surgically}}\times \frac{\mathrm{Level}\ \mathrm{of}\ \mathrm{diagnostic}\ \mathrm{confidence}\ \mathrm{above}\ \mathrm{which}\ \mathrm{decison}\ \mathrm{of}\ \mathrm{colectomy}\ \mathrm{was}\ \mathrm{taken}\ }{1-\mathrm{Level}\ \mathrm{of}\ \mathrm{diagnostic}\ \mathrm{confidence}\ \mathrm{above}\ \mathrm{which}\ \mathrm{decison}\ \mathrm{of}\ \mathrm{colectomy}\ \mathrm{was}\ \mathrm{taken}}\right) $$

### Statistical analysis

InStat 3.01, GraphPad Software, San Diego, CA, USA, was used for statistical analysis purposes. Categorical variables are presented as frequency (percentages) and continuous and ordinal variables are presented as mean ± standard deviation (SD). The consistency between the radiological and the surgical-pathological stages for TN stage and the consistency between CTC or colonoscopy and surgery for tumor location were estimated with the weighted kappa or kappa (where applicable) coefficient (*κ*) at 95% confidence interval (CI) [4]. The results were considered significant at 95% of the confidence level.

## Results

### Demographical and clinical conditions

All included patients had adenocarcinomas, and in 11 (4%) of patients, synchronous cancer was detected. Therefore, a total of 280 tumors were evaluated by colonoscopy, CTC, exercised surgically, and examined surgical pathologically. Patients had 40 to 70 years of age range at the time of diagnosis of colon cancer. The other clinical conditions of the enrolled patients are reported in the Table 2.

**Table 2:** Demographic and clinical conditions of the patients at the time of diagnosis

Parameters		Value
Numbers of patients included in the analysis		269
Numbers of tumors evaluated for the analysis		280
Sex	Male	158(59)
	Female	111(41)
Age (years)	Minimum	40
	Maximum	70
	Mean ± SD	58.42 ± 9.15
The time between the computed tomography colonography and colectomies (days)		30 ± 11

Categorial variables are presented as frequency (percentages) and continuous variables are presented mean ± standard deviation (SD)

### Colectomy

After colonoscopy, CTC examinations changed the colectomy plan in 79 (28%) patients (72 (26%) patients due to wrong localization of tumor by colonoscopy, and 7 (2%) patients due to synchronous tumors).

### Location of tumor

All tumors detected during colonoscopy and CTC were correctly identified during surgeries. According to surgical location (reference standard) of tumors, tumor locations prediction by colonoscopy and CTC with errors are presented in Table 3. CTC detected all tumors (*n* = 280) and reported correct location of tumors in 261 (93%) cases. CTC made a mistake in the location of tumor by one contiguous segment in 19 (7%) cases. CTC did not make a mistake in the location of tumor by more than one contiguous segment. The sensitivity and accuracy of CTC for the detection of the location of colon tumors were 100% and 92.58%. The *κ* value for tumor location between surgeries and CTC was 0.89 (95% Cl: 0.83–0.95). Colonoscopy detected 273 (98%) tumors but did not detect 7 (2%) tumors due to stenosis tumors because these did not allow passage of endoscopy (synchronous tumors). Colonoscopy reported correct location of tumors in 201 (72%) and made a mistake in the location of tumors by one contiguous segment in 55 (20%) tumors and made a mistake in the location of tumors by more than one contiguous segment in 17 (6%) cases. The *κ* value for tumor location between surgeries and colonoscopy was 0.65 (95% Cl: 0.54–0.72). The sensitivity and accuracy of colonoscopy for the detection of the location of colon tumors were 96.63% and 71.79%.

**Table 3:** Prediction of locations of tumors in different colon segments by index tests with errors

Prediction of locations of tumors	Index tests					
Colon segments	Colonoscopy			The computed tomography colonography		
Values	Correct	Error, 1 segment	Error, > 1 segment	Correct	Error, 1 segment	Error, > 1 segment
Tumors evaluated	280	280	280	280	280	280
Sigmoid colon	105 (38)	8 (3)	0 (0)	116 (41)	0 (0)	0 (0)
Caecum	36 (13)	0 (0)	0 (0)	36 (13)	0 (0)	0 (0)
Ascending colon	26 (9)	12 (4)	3 (1)	41 (15)	4 (1)	0 (0)
Descending colon	12 (4)	8 (3)	3 (1)	20 (7)	2 (1)	0 (0)
Hepatic flexure	10 (4)	8 (3)	3 (1)	14 (5)	8 (3)	0 (0)
Transverse colon	5 (2)	6 (2)	6 (2)	14 (5)	3 (1)	0 (0)
Splenic flexure	7 (2)	13 (5)	2 (1)	20 (7)	2 (1)	0 (0)
Prediction of locations of tumors in all colon segments	201 (72)	55 (20)	17 (6)	261 (93)	19 ( (7)	0 (0)

Surgical locations of tumors were considered reference standard

Variables are presented as frequency (percentages)

### Tumor staging

The T and N stages of tumors according to surgical pathology are reported in Table 4. CTC was correctly staged 193 lesions for T staging (Table 5). The *κ* value for T staging of tumors between CTC and surgical pathology was 0.65. The accuracy of CTC for differentiation of tumors confined to the colon wall (T1/T2) from advanced tumors (T3/T4) was 83.57%. The *κ* value for differentiation of tumors confined to the colon wall (T1/T2) from advanced tumors (T3/T4) between CTC and surgical pathology was 0.69 (Table 6). For classification of tumors between low–intermediate risk (T1/T2 and T3ab) and high risk (T3cd and T4), the accuracy of CTC was 83.93%. *κ* value for classification of tumor between low–intermediate risk and high risk between CTC and surgical pathology was 0.68 (Table 7). The accuracy of CTC for the involvement of the colonic circumference was 82.14%. The *κ* value for the involvement of the colonic circumference between CTC and surgical pathology was 0.67 (Table 8).

**Table 4:** The T and N stages of tumors according to surgical pathology

T stage		Number (percentages)	N+ (positive for lymph node metastases)	Length (cm)
Tumors evaluated for the analysis		280	280	280
pT1–2		109 (39)	5 (2)	2.71 ± 1.12
pT3	< 5 mm (pT3ab)	70 (25)	21 (8)	3.55 ± 1.92
	≥ 5 mm (pT3cd)	29 (10)	14 (5)	3.44 ± 1.85
pT4a		59 (21)	40 (14)	4.05 ± 1.15
pT4b		13 (5)	8 (3)	5.01 ± 2.01
Total		280 (100)	88 (32)	N/A

Categorical variables are presented as frequency (percentages) and continuous variables are presented mean ± standard deviation (SD)

*N/A* not applicable

**Table 5:** Results of the computed tomography colonography and surgical pathology for predicting of T stage of tumor

The T stage	The T stage according to the surgical pathology						Comments on the prediction of T stage by CTC according to the results of to the surgical pathology		
The prediction of T stage according to CTC	pT1/T2	pT3ab	pT3cd	pT4a	pT4b	Total	Over-staged	Under-staged	Correct
cT1/T2	79 (28)	12 (4)	01 (0.5)	03 (1)	0 (0)	95 (33.5)	–	16 (5.5)	79 (28)
cT3ab	10 (4)	44 (16)	03 (1)	03 (1)	01 (0.5)	61 (22.5)	10 (4)	7 (2.5)	44 (16)
cT3cd	09 (3)	06 (2)	22 (7.5)	11 (4)	01 (0.5)	49 (17)	15 (5)	12 (4.5)	22 (7.5)
cT4a	08 (3)	07 (3)	03 (1)	39 (14)	02 (1)	59 (22)	18 (7)	02 (1)	39 (14)
cT4b	03 (1)	00 (0)	00 (0)	03 (1)	9 (3)	15 (5)	6 (2)	–	9 (3)
Total	109 (39)	70 (25)	29 (10)	59 (21)	13 (5)	280 (100)	49 (18)	37 (13.5)	193 (68.5)

Variables are presented as frequency (percentages)

*CTC* computed tomography colonography

**Table 6:** Results of the computed tomography colonography and surgical pathology for predicting for the differentiation of tumors confined to the colon wall (T1*/*T2) from advanced tumors (T3*/*T4)

Differentiation of tumors T1/T2 stage from T3/T4 stage	Differentiation of tumors T1/T2 stage from T3/T4 according to the surgical pathology			Comments on the prediction of differentiation of tumors T1/T2 stage from T3/T4 by CTC^a^		
The prediction of differentiation of tumors T1/T2 stage from T3/T4 according to CTC	Tumors confined to the colon wall (T1/T2)	Locally advanced tumors (T3/T4)	Total	Over-staged	Under-staged	Correct
Tumors confined to the colon wall (T1/T2)	79 (28)	16 (5.5)	95 (33.5)	–	16(5.5)	79 (28)
Locally advanced tumors (T3/T4)	30 (11)	155 (55.5)	175 (66.5)	30 (11)	–	155 (55.5)
Total	109 (39)	171 (61)	280 (100)	30 (11)	16 (5.5)	234 (83.5)

Variables are presented as frequency (percentages)

*CTC* computed tomography colonography

^a^According to the results of to the surgical pathology

**Table 7:** Results of the computed tomography colonography and surgical pathology for predicting for the differentiation of tumors between low–intermediate risk (T1/T2 and T3ab) and high risk (T3cd and T4)

Differentiation of tumors between low–intermediate risk and high risk	Differentiation of tumors between low–intermediate risk and high risk according to the surgical pathology			Comments on the prediction of differentiation of tumors between low–intermediate risk and high risk by CTC according to the results of to the surgical pathology		
The prediction of differentiation of tumors between low–intermediate risk and high risk according to CTC	Low–intermediate risk (T1/T2 and T3ab) tumor	High risk (T3cd and T4) tumor	Total	Over-staged	Under-staged	Correct
Low-intermediate risk (T1/T2 and T3ab) tumor	145 (52)	11 (4)	156 (56)	–	11 (4)	145 (52)
High risk (T3cd and T4) tumor	34 (12)	90 (32)	124 (44)	34 (12)	–	90 (32)
Total	179 (64)	101 (36)	280 (100)	34 (12)	11 (4)	235 (84)

Variables are presented as frequency (percentages)

*CTC* computed tomography colonography

**Table 8:** Results of the computed tomography colonography and surgical pathology for predicting for the involvement of the colonic circumference

The T stage according to the colonic circumference involvement	The colonic circumference involvement according to the surgical pathology			Comments on the prediction of colonic circumference involvement by CTC according to the results of to the surgical pathology		
The prediction of colonic circumference involvement according to CTC	pT1/T2	pT3-T4	Total	Over-staged	Under-stage	Correct
< 50%/ < 180°	94 (34)	35 (12)	129 (46)	–	35 (12)	94 (34)
≥ 50%/ ≥ 180°	15 (5)	136 (49)	151 (54)	15 (5)	–	136 (49)
Total	109 (39)	171 (61)	280 (100)	15 (5)	35 (12)	230 (83)

Variables are presented as frequency (percentages)

*CTC* computed tomography colonography

### Node staging

The count for median lymph node was 19.95 ± 8.89 (the range: 5–76)/tumor. A total of 88 (32%) had nodal metastasis (N+). Sensitivity, specificity, and accuracy for CTC for the N stage of the tumor were 69.31%, 66.15%, and 67.14% respectively. The *κ* value for T staging of tumors between CTC and surgical pathology was 0.41 (Table 9).

**Table 9:** Results of the computed tomography colonography and surgical pathology for predicting of the node staging

The N stage	The N stage according to the surgical pathology			Comments on the prediction of N stage by CTC according to the results of to the surgical pathology		
The prediction of N stage according to CTC	pN+	pN-	Total	Over-staged	Under-stage	Correct
cN+	61 (22)	65 (23)	136 (45)	65 (23)	–	61 (22)
cN-	27 (10)	127 (45)	154 (55)	–	27 (10)	127 (45)
Total	88 (32)	192 (68)	280 (100)	65 (23)	27 (10)	188 (67)

Variables are presented as frequency (percentages)

*CTC* computed tomography colonography

### Diagnostic parameters

The different diagnostic parameters for predicting the T and N stages of tumors are reported in Table 10.

**Table 10:** Diagnostic parameters of the computed tomography colonography for predicting the T and N stages of tumors of the colon

Prediction of stage of tumor	True Positives (TP)	True Negative (TN)	Positive predictive value (PPV) (TP + TN)	False Positives (FP)	False Negative (FN)	Negative predictive value (NPV) (FP + FN)	Sensitivity	Specificity	Accuracy	*κ*^a^	Cl^a^
T staging	193 (68.5)	01 (0.5)	194 (69)	37 (13)	49 (18)	86 (31)	79.75%	2%	69.29%	0.65	0.53–0.72
Differentiation of tumors confined to the colon wall (T1/T2) from advanced tumors (T3/T4)	79 (28)	155 (55.5)	234 (83.5)	16 (5.5)	30 (11)	46 (16.5)	72.48%	90.64%	83.57%	0.69	0.51–0.75
Differentiation of tumors between low–intermediate risk and high risk	145 (52)	90 (32)	235 (84)	11 (4)	34 (12)	45 (16)	81.01%	89.11%	83.93%	0.68	0.53–0.75
Involvement of the colonic circumference	94 (34)	136 (49)	230 (83)	35 (12)	15 (5)	50 (17)	86.24%	79.53%	82.14%	0.67	0.54–0.71
N staging	61 (22)	127 (45)	188 (67)	65 (23)	27 (10)	92 (33)	69.31%	66.15%	67.14%	0.41	0.59–0.69

Variables are presented as frequency (percentages)

*Cl* confidence interval

*κ* weighted kappa or kappa coefficient

^a^Between the computed tomography colonography and surgical pathology

### Beneficial score analysis

**Fig. 8:**
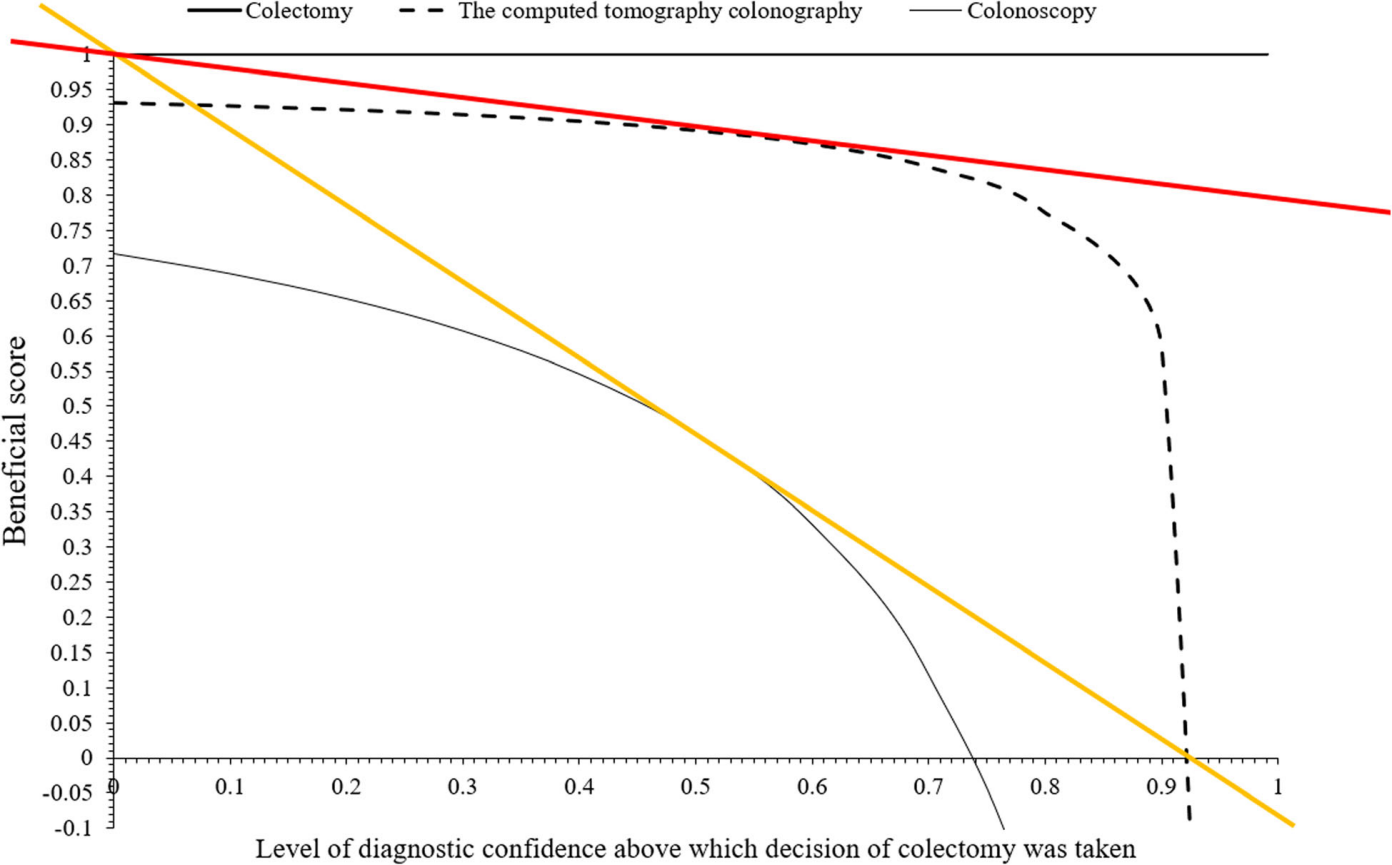
Beneficial score analysis for index tests. Image analysis was performed by radiologists (a minimum of 10 years of experience in abdominal computed tomography) of institutes. Pathologies were performed by pathologists (a minimum of 3 years of experience; unaware of the computed tomography colonography findings) of institutes. The area under the orange line is the correct location of the tumor without mistake by one or more than one contiguous segment. The area between the orange line and the red line is the location of the tumor with a mistake by more than one contiguous segment. The area above the red line is the location of the tumor with a mistake by one contiguous segment

Beneficial scores for CTC and colonoscopy were 0–0.921 diagnostic confidence and 0–0.734 diagnostic confidence. Above 0.921 diagnostic confidence, CTC and colonoscopy reported an error for the location of colon tumors by one contiguous segment, and above 0.734 diagnostic confidence, colonoscopy reported an error for the location of colon tumors by more than one contiguous segment (Fig. 8; Suppl. Table 1).

## Discussion

The study reported that the sensitivity and accuracy of CTC for detection of the location of colon tumors were 100% and 92.58%. Also, CTC did not make a mistake in the location of tumors by more than one contiguous segment and had a higher beneficial score for the surgical procedure of colon tumors than colonoscopy (0–0.921 diagnostic confidence vs. 0–0.734 diagnostic confidence). The results of the sensitivity and accuracy of CTC for detection of the location of colon tumors of the current study agreed with those of the prospective studies [4–6] and a comparative study [7]. The current study divided the colon into seven segments including hepatic and splenic features as separate segments similar to the prospective study on the Spanish population [4]. The different studies [5–7, 20, 21] divided the colon into five segments to decrease errors. CTC have high sensitivity and accuracy than colonoscopy for the localization of colon tumors.

Sensitivity and accuracy of colonoscopy for detection of the location of colon tumors were 96.63% and 71.79%. Also, colonoscopy did not detect 7 (2%) tumors due to stenosis tumors, made a mistake in the location of tumors in 72 (26%) cases, and had fewer beneficial scores for the surgical procedure of colon tumors than CTC. The results of the sensitivity and accuracy of colonoscopy for detection of the location of colon tumors of the current study agreed with those of the prospective studies [4, 5] and comparative studies [8, 9, 12, 22]. A colorectal surgeon even with a minimum of 10 years of experience needs the exact location of the colon tumor during laparoscopy because palpation is not possible for the colon [4, 22]. Difficulty in the identification of the lesion during colectomy may lead to a change in the resection than originally planned [12]. The pre-colectomy colonoscopy findings for colon cancer do not provide the exact location of the colon tumor and have chances of difficulties during colectomy.

The study reported 72.48% sensitivity, 90.64% specificity, and 83.57% accuracy of CTC for differentiation of tumors confined to the colon wall (T1/T2) from advanced tumors (T3/T4). The results of diagnostic parameters for differentiation of tumors confined to the colon wall from advanced tumors of the current study agreed with those of prospective study [4] and a retrospective study [23]. CT has the ability to detect invasion of tumors beyond the bowel wall (T1/T2 vs. T3/T4) [1] but CT has poor diagnostic parameters for differentiation of tumors confined to the colon wall from advanced tumors [24]. The distension of the colon in CTC improves the evaluation of the wall of the colon [4]. CTC is a good choice for differentiation of tumors confined to the colon wall (T1/T2) from advanced tumors (T3/T4).

Patients were examined on a 64-slice multidetector CT scanner with thin reconstruction and MPR images. Direct signs of local invasion for T staging, stranding of fat and spiculation, or thickening of visceral serosa can be found due to desmoplastic reaction [4]. A multidetector scanner with thin reconstruction and MPR images allows accurate T staging than the evaluation of direct signs.

CTC was performed after air distension of the colon. Recently, CTC is performed using water as the endoluminal contrast [21, 25, 26]. Water requires good anal continence that is not possible in elderly patients. Also, these studies are performed with a small sample size. Therefore, the current study performed CTC after air distension of the colon.

The study reported 81.01% sensitivity, 89.11% specificity, and 83.93% accuracy of CTC for differentiation of tumors between low–intermediate risk and high risk. The results of CTC for differentiation of tumors between low–intermediate risk and high risk of the current study agreed with those of the prospective study [4] and a retrospective study [27]. CTC after air distension of the colon provided more accurate results.

The study reported 69.31% sensitivity, 66.15% specificity, and 67.14% accuracy of CTC for N staging of tumors but did not differentiate nodes in benign and metastatic. The results of CTC for N staging of the current study are agreed with those of the prospective study [4]. CTC is not successful for the selection of patients for neoadjuvant chemotherapy.

The work is interesting for the field but has some limitations, for example, a small clinical retrospective analysis and a lack of prospective study. A lack of comparisons of the TNM staging and tumor risk in which sensitivity, specificity, and accuracy with conventional CT analysis. The time between CTC and colectomy was quite long (30 ± 11 days), which may progress tumor in the patients. CTC is not allowed biopsies. A diagnostic performance study is required including patients with colon cancer who received neoadjuvant chemotherapy (future study).

## Conclusions

In patients with colon cancer, accurate preoperative evaluation is essential for a correct therapeutic plan. The computed tomography colonography has improved diagnostic parameters for pre-colectomy location and T staging of colon tumors. However, the study results showed moderate interobserver variability in radiologists. In addition, the study result shows that the computed tomography colonography has high sensitivity but low specificity for tumor staging and localization, and suspicible to different readers. The preoperative computed tomography colonography findings for colon cancer optimized the surgical management plan but did not provide information for the selection of neoadjuvant chemotherapy or not.

## Supplementary Information

**Additional file 1: Suppl. Table 1**. Beneficial score analysis for index tests.

## Data Availability

The datasets generated during and/or analyzed during the current study are not publicly available, but are available from the corresponding author on reasonable request.

## Abbreviations

Cl: Confidence interval
cN: The N stage according to the computed tomography colonography
cT: The T stage according to the computed tomography colonography
CT: Computed tomography
CTC: Computed tomography colonography
FN: False negative
FP: False positive
*κ*: Weighted *kappa* or *kappa* coefficient
MPR: Multiplanar reformation
NPV: Negative predictive value
pN: The N stage according to the surgical pathology
PPV: Positive predictive value
pT: The T stage according to the surgical pathology
SD: Standard deviation
TN: True negative
TNM: Tumor, node, and metastasis
TP: True positive
