# Supplementary material for: Pre-colectomy location and TNM staging of colon cancer by the computed tomography colonography: a diagnostic performance study
Source: World J Surg Oncol. 2021 Apr 15;19:120. doi: 10.1186/s12957-021-02215-4 (PMC8051039; doi:10.1186/s12957-021-02215-4)
Supplement: Supplementary file 1 — Additional file 1: Suppl. Table 1. Beneficial score analysis for index tests. [file 12957_2021_2215_MOESM1_ESM.docx]

Suppl. Table 1. Beneficial score analysis for index tests.

| Level of diagnostic confidence above which decision of colectomy was taken | Beneficial score | | |
| --- | --- | --- | --- |
|  | Colectomy | The computed tomography colonography | Colonoscopy |
| 0 | 1 | 0.932143 | 0.717857 |
| 0.1 | 1 | 0.927778 | 0.689286 |
| 0.2 | 1 | 0.922321 | 0.653571 |
| 0.3 | 1 | 0.915306 | 0.607653 |
| 0.4 | 1 | 0.905952 | 0.546429 |
| 0.5 | 1 | 0.892857 | 0.460714 |
| 0.6 | 1 | 0.873214 | 0.332143 |
| 0.7 | 1 | 0.840476 | 0.117857 |
| 0.8 | 1 | 0.775 | -0.31071 |
| 0.9 | 1 | 0.578571 | -1.59643 |
| 0.99 | 1 | -2.95714 | -24.7393 |

0: No diagnostic confidence; 0.99: Maximum diagnostic confidence
